# Effect of Moxibustion Treatment on Degree Centrality in Patients With Mild Cognitive Impairment: A Resting-State Functional Magnetic Resonance Imaging Study

**DOI:** 10.3389/fnhum.2022.889426

**Published:** 2022-08-02

**Authors:** Ke Xu, Yichen Wei, Chengxiang Liu, Lihua Zhao, Bowen Geng, Wei Mai, Shuming Zhang, Lingyan Liang, Xiao Zeng, Demao Deng, Peng Liu

**Affiliations:** ^1^Life Science Research Center, School of Life Sciences and Technology, Xidian University, Xi’an, China; ^2^Engineering Research Center of Molecular and Neuro Imaging Ministry of Education, School of Life Sciences and Technology, Xidian University, Xi’an, China; ^3^Department of Radiology, The People’s Hospital of Guangxi Zhuang Autonomous Region, Nanning, China; ^4^Department of Acupuncture, First Affiliated Hospital, Guangxi University of Chinese Medicine, Nanning, China

**Keywords:** mild cognitive impairment, moxibustion treatment, rs-fMRI, degree centrality, cognitive function

## Abstract

**Background:**

Mild cognitive impairment (MCI) is a common neurological disorder. Moxibustion has been shown to be effective in treating MCI, but its therapeutic mechanisms still remain unclear. This study mainly aimed to investigate the modulation effect of moxibustion treatment for patients with MCI by functional magnetic resonance imaging (fMRI).

**Methods:**

A total of 47 patients with MCI and 30 healthy controls (HCs) participated in resting-state fMRI imaging (rs-fMRI) scans. Patients with MCI were randomly divided into true moxibustion group (TRUE, *n* = 30) and sham moxibustion group (SHAM, *n* = 17). The degree centrality (DC) approach was applied to distinguish altered brain functions. Correlation analysis was then performed to examine the relationships between the neuroimaging findings and clinical symptoms.

**Results:**

Compared with HCs, patients with MCI mainly showed decreased DC in the left middle frontal cortex (MFC) and bilateral middle cingulate cortex (MCC). After moxibustion treatment, the SHAM group had no significant DC findings, while TRUE group mainly showed significant increased DC in the bilateral MFC and MCC, as well as decreased DC in the left middle occipital cortex (MOC). Repeated measures analysis of variance (ANOVA) showed significant interactions between the two groups of patients with MCI. In addition, the higher Mini-Mental State Examination (MMSE) score was significantly positively correlated with increased DC in the right MFC and left MCC after moxibustion treatment.

**Conclusion:**

Our findings demonstrate that the potential value of moxibustion treatment on MCI, which adds new insights into the popular view that moxibustion treatment may slow cognitive decline in patients with MCI.

## Introduction

Mild cognitive impairment (MCI) is a condition characterized by memory problem and is the prodromal form of Alzheimer’s disease (AD) but not to the degree of dementia. MCI signifies the transitional stage between healthy aging and dementia. According to the reports, almost half of patients with MCI tend to progress to fulfill diagnostic criteria of dementia within 5 years (Petersen et al., 2001; Wentzel et al., 2001). Therefore, the improvement of MCI can delay the onset of AD, and it may be an important treatment strategy for AD. Nevertheless, there are no recommended medications for MCI because cholinesterase inhibitors, the well-known antidementia drugs, have more adverse effects than benefits when prescribed to patients with MCI (Russ and Morling, 2012; Vega and Newhouse, 2014). It seems that pharmacological treatment for MCI is far from satisfactory (Petersen et al., 2018), and effective non-pharmacological treatment has gained considerable attention in recent years. Recently, researchers and doctors tend to use complementary and alternative therapies including traditional Chinese medicine (TCM) to treat MCI, and TCM therapy may play a persistent role in relieving cognitive impairment in patients with MCI.

As a non-drug treatment, moxibustion treatment is unique. Moxibustion treatment is easy to operate and does not require professional training for patients and is one of the categories of TCM therapy that has been widely used in East Asia for thousands of years (Shen et al., 2006). Moxibustion treatment imparts both heat stimulation *via* infrared radiation and the pharmacological actions of its herbal components (Kawakita et al., 2006; Okada and Kawakita, 2009). It regulates a multidimensional network that includes the nervous, endocrine, and immune systems, all of which play important roles in maintaining homeostasis, potentially exerting significant therapeutic effect (Huang et al., 2017). Moxibustion treatment has the advantages of non-toxic, no side effects, low cost, remarkable curative effect, convenient and quick, no pain, and so on, making it an optimal choice for older individuals with cognitive decline. Various clinical trials and animal studies have been conducted to investigate the benefits and mechanisms of moxibustion for preventing and treating MCI (Choe et al., 2018; Zhang et al., 2020). It has been reported that moxibustion treatment might improve the clinical symptoms and regulate neuropeptides related to learning and memory in patients with dementia (Wang et al., 2010; Chen et al., 2011). Studies have shown that moxibustion treatment can improve cognitive function in patients with MCI and AD (Wang et al., 2020; Zhang et al., 2020). However, probing the alleviation of cognitive function impairment in patients with MCI by moxibustion treatment with classic measurements and investigating its underlying neurobiological mechanisms are still lack.

In recent years, resting-state functional magnetic resonance imaging (rs-fMRI) has gained increasing attention for studying the neural mechanisms of cognitive dysfunction in patients with MCI (Wang et al., 2011; Lau et al., 2016; Zhen et al., 2018; Soman et al., 2020), and many rs-fMRI analysis algorithms have been applied to the research of MCI. For instance, based on metrics that reflect regional spontaneous neuronal activity such as regional homogeneity (ReHo) and the amplitude of low-frequency fluctuation (ALFF), cognitive impairment in amnestic patients with MCI (aMCI) has been associated significantly with decreases in ALFF in the cuneus/precuneus cortices (Pan et al., 2017). A meta-analysis has identified significant ReHo alterations in patients with aMCI relative to healthy controls (HCs), mainly within bilateral middle temporal gyri, left parahippocampus/hippocampus, dorsolateral prefrontal cortex, and left middle occipital gyrus (Zhen et al., 2018). Thereby, rs-fMRI has been proven to effectively evaluate the pathological mechanism of MCI, which may indicate the potential value of rs-fMRI to evaluate the relevant treatment effect of moxibustion treatment on MCI.

In this study, we applied voxel-wise degree centrality (DC) to assess distinguished patterns of brain intrinsic connectivity between patients with MCI and HCs, such as node importance within the whole-brain network. DC approach can quantify the importance of each node in the brain network and allows the mapping of functional integration in the brain at the voxel level. More importantly, we tried to investigate the effect of moxibustion treatment in patients with MCI and possible correlations between the neuroimaging findings and changes of MCI-related clinical symptoms after moxibustion treatment. In this study, our hypothesis was that moxibustion treatment would attenuate DC-related progression of cognitive deterioration in patients with MCI. Specifically, we expected amelioration in the memory, executive function, and attention domain in patients with MCI after moxibustion treatment.

## Materials and Methods

### Participants

Totally, 47 patients with MCI and 30 HCs were recruited. Patients were included based on the criteria: (a) memory disorder as the chief complaint, which was corroborated by an informant; (b) relatively intact or slightly impaired of general cognitive function; (c) having no influence on daily living activity; (d) failure of meeting the diagnostic criteria for dementia; (e) having no other systemic diseases that might cause brain function decline; (f) the Global Deteriorate Scale (GDS) score: 2–3, and the Clinical Dementia Rating (CDR) score: 0.5. Exclusion criteria were as follows: (a) other advanced, severe, or unstable diseases, such as liver, kidney, and serious primary diseases; (b) severely impaired of hearing and vision, failure of cooperating with assessment; (c) patients with active epilepsy; (d) having a history of mental illness; (e) having dementia, cerebral infarction, or any physical and mental disorders that can bring about cerebral dysfunction; (f) taking medications that may cause changes in cognitive function or failure of vital organs, such as the heart, brain, and kidneys; (g) patients aged under 55 or over 75 years; (h) contraindications for MRI examination; (i) left- and double handedness; and (j) those who were unable to cooperate with the completion of the corresponding evaluation and inspection and quit halfway.

### Moxibustion Treatment

**FIGURE 1:**
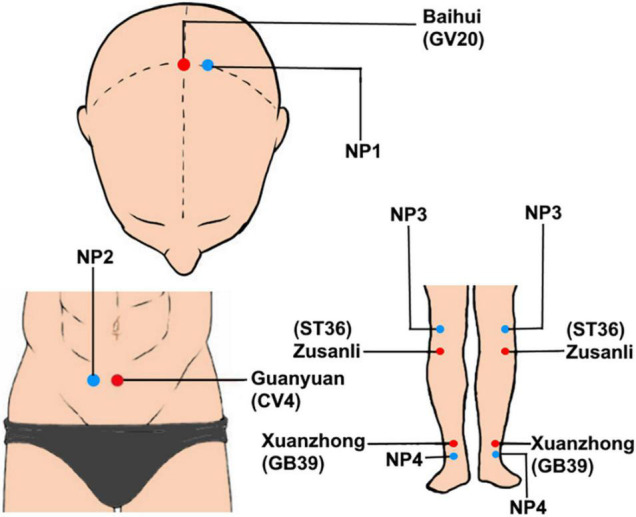
Locations of selected acupoints and non-acupoints. The acupoints were Baihui (GV20), Guan yuan (CV4), bilateral Zusanli (ST36), and Xuanzhong (GB39).

According to previous studies on the treatment of cognitive disorders with moxibustion (Aum et al., 2021) and *Acupuncture* and *Moxibustion* compiled by Shi Xuemin (Shi, 2007), we selected six acupoints for true moxibustion group (TRUE), including Baihui (GV20), Guan yuan (CV4), bilateral Zusanli (ST36), and Xuanzhong (GB39). These acupoints are important for cognitive modulation, for example, Baihui (GV20) is applied in the treatment to alleviate neurodegenerative disorders and cognitive impairment (Zhu and Zeng, 2011; Lee et al., 2014). GV20 and ST36 are used to improve cognitive function by enhancing the plasticity of the hippocampus (Ye et al., 2017). Non-acupoints are selected as control points near the true acupoints of moxibustion. The selected non-acupoints in the sham moxibustion group (SHAM) include NP-1 [3.33 cm above left Shuaigu (GB8) on the head], NP-2 (10 cm to the right of Guan yuan), NP-3 (bilateral inferior margin of patella), and NP-4 (3.33 cm above bilateral Waihuaijian). Both six acupoints in true moxibustion group (TRUE) and non-acupoints in the sham moxibustion group (SHAM) are shown in Figure 1. Following training, the acupuncturists with more than 5 years of clinical experience were in charge of manipulating. The double acupoints were taken on both sides each time. During the moxibustion treatment, vaseline was applied to the acupoints to protect the patient’s skin and facilitate the fixation of moxa-cones. Each moxa-cone has a diameter of 1.5 cm, a height of 3 cm, and a weight of 5 g. One moxa-cone was burnt about 10–15 min each time for each acupoint. Patients with MCI received treatment every other day, 15 times for 1 course, and rested for 3 days to continue the next course, for a total of 2 courses of treatment.

### Cognitive Function Measurement

We used the Montreal Cognitive Assessment (MoCA) and Mini-Mental State Examination (MMSE), and cognitive screening instruments created and validated to detect MCI, as our primary outcome measure. MoCA is a brief (about 10 min) screening tool for MCI that evaluates visual space, executive function (clock drawing), naming, attention, language, abstract ability, memory, and orientation with a total score of 0–30 (Yu et al., 2012; Zheng et al., 2016). The MMSE evaluates orientation, memory, calculation and attention, recall and language (Folstein et al., 1975), which has become the well-known and the most often used short screening tool for providing an overall measure of cognitive impairment in clinical, research, and community settings. The higher score of MoCA and MMSE represents the better cognitive function. The MoCA and MMSE were measured at baseline and after 2-month treatment.

### MRI Data Acquisition

The MRI data were acquired on a 3.0 T Siemens Magnetom Verio MRI System (Siemens Medical, Erlangen, Germany), using a standard head coil. Participants were requested to keep their eyes closed and awake, and remain still without thinking about anything. Foam pillows were used for minimizing movement between the instrument and each participant’s head. The rs-fMRI data were collected by a single-shot gradient-recalled echo planar imaging (EPI) sequence: repetition time (TR) = 2,000 ms; echo time (TE) = 30 ms; flip angle (FA) = 90°; field of view (FOV) = 240 mm × 240 mm; matrix size: 64 × 64; slice thickness = 5 mm (no-gap); 31 slices; and 180 volumes. High-resolution T1-weighted images were then obtained with a magnetization-prepared rapid acquisition gradient echo sequences with the following parameters: TR = 1,900 ms; TE = 2.22 ms, FOV = 250 mm × 250 mm, matrix size: 256 × 256, FA = 9°, slice thickness = 1 mm and 176 slices.

### MRI Data Preprocessing

MRI data included the 6 min-resting data from HCs and patients with MCI at baseline, as well as the 6 min-resting data from patients with MCI at the end of moxibustion treatment. Based on Statistical Parametric Mapping 12 (SPM12, United Kingdom^1^) on the MATLAB platform, the preprocessing of rs-fMRI imaging data was conducted by Data Processing Analysis of Brain Imaging (DPABI 4.3^2^) (Yan et al., 2016). The first five volumes of functional data for each subject were discarded for signal equilibrium and subject adaptation to the imaging noise. The remaining volumes were slice-timing corrected and head-motion corrected. After realignment, all images were normalized to the standard Montreal Neurological Institute (MNI) template and then resampled into 3 mm × 3 mm × 3 mm resolution. The 24-head motion parameters and average signals from the white matter and cerebrospinal fluid (CSF) were used as nuisance covariates to reduce the effects of head motion and non-neuronal blood oxygenation level-dependent (BOLD) fluctuations. To reduce low-frequency drift and high-frequency respiratory and heart rhythms, the linear trend in the fMRI data was removed, and the images were temporally band-pass filtered (0.01–0.1 Hz).

### Degree Centrality Calculation

The preprocessed fMRI data were used for DC calculation, and voxel-wise DC calculations were performed using the DPABI software. We extracted the BOLD time series of each voxel and computed Pearson’s correlation coefficients (*r*) between any pair of brain voxels within the whole-brain gray matter mask. Owing to the uncertainty of interpretation and detrimental effects on test-retest reliability, only positive Pearson correlation coefficients were considered in the DC calculations. For each voxel, *i*, the number of strong voxel-to-voxel correlations (defined as correlation coefficient *r* > 0.25) was computed to determine the DC of *i*. The correlation threshold *r* was set at 0.25 in accordance with previous studies (Buckner et al., 2009; Zuo et al., 2012). The weighted DC value of a voxel was calculated and a map of DC values was obtained for each participant. To improve normality, Fisher’s *r*-to-*z* transformation was used to obtain the *z*-score DC map. Prior to statistical analysis, all individual DC maps were spatially smoothed with a Gaussian smoothing kernel (full-width half maximum, FWHM = 6 mm).

### Statistical Analysis

Statistical differences that were not related to voxel computations were calculated using the SPSS software (version 22.0; IBM, Armonk, NY, United States). Continuous data normality within sub-groups was assessed using the Shapiro–Wilk test. Differences between patients with MCI and HCs regarding demographical data (i.e., age, education, gender, pre-MMSE, pre-MoCA, MMSE, and MoCA) were calculated as follows: continuous, normally distributed variables were subjected to the two-sample *t*-test; continuous, non-normally distributed variables were analyzed using the Mann–Whitney *U*-test and Wilcoxon signed-rank test while the Chi-square test was used for categorical variables. The significance threshold was set at *P* < 0.05 for all analysis.

A two-sample *t*-test was applied to examine differences in DC between patients with MCI and HCs with ages, education, and gender as the covariances. The paired *t*-test was then applied to explore the DC intra-group changes in two groups of patients with MCI before and after moxibustion treatment. A threshold of voxel-wise *P* < 0.005 uncorrected with cluster-level *P* < 0.05 false discovery rate (FDR) corrected was applied for the *t*-test analysis.

On the base of regions showing significant paired *t*-test group differences, five regions of interest (ROIs) were selected from the sets of voxels within 6 mm spheres with centers at the peak of the clusters, and repeated measures analysis of variance (ANOVA) was used to further explore the DC between-group differences in two groups of patients with MCI before and after moxibustion treatment. Pearson’s correlation analysis was used to investigate whether the mean DC changes (posttreatment minus pretreatment) in each ROI correlated with the changes (posttreatment minus pretreatment) in MoCA and MMSE score for patients with MCI (*P* < 0.05, Bonferroni corrected).

## Results

### Demographics and Clinical Results

A total of 77 participants were enrolled in this study, including 47 patients with MCI and 30 matched HCs. There were no significant differences between the two groups in terms of age, education, and gender (*P* > 0.05). Compared with HCs, patients with MCI exhibited significantly lower in MMSE and MoCA score (*P* < 0.05). Their demographic and neuropsychological data are summarized in Table 1. Notably, 47 patients with MCI were randomly divided into TRUE (*n* = 30) and SHAM (*n* = 17) groups (Table 2). After moxibustion treatment, increased MMSE and MoCA score (*P* < 0.05) in two groups of patients with MCI are exhibited in Table 3. TRUE group had increased MMSE score (mainly including attention and calculation: 1.27 ± 1.28; recall: 0.80 ± 0.96) and increased MoCA score (mainly, including clock drawing: 0.47 ± 1.04; attention: 0.83 ± 1.05; memory: 2.07 ± 1.53) after moxibustion treatment. SHAM group also showed increased MMSE (mainly, including attention and calculation: 0.65 ± 1.9; recall: 0.50 ± 0.87) and increased MoCA score (mainly including clock drawing: 0.45 ± 0.92; attention: 0.55 ± 1.07; memory: 1.5 ± 1.07) after moxibustion treatment, albeit at a lesser extent.

**TABLE 1:** Demographic and clinical conditions in patients with mild cognitive impairment (MCI) and healthy controls (HCs).

Characteristic	MCI (*n* = 47)	HCs (*n* = 30)	*P*-value
Age (years)	64.3 ± 6.5	66.1 ± 5.9	0.234^a^
Gender (M/F)	14/33	13/17	0.224^b^
Education (years)	11 (8∼14)^d^	14 (8∼15)^d^	0.107^c^
Pre-MMSE	26 (25∼27)^d^	29 (28∼30)^d^	0.000^c^
Pre-MoCA	21.9 ± 2.7	25.6 ± 2.1	0.000^a^

*Pre-MMSE, MMSE before moxibustion treatment; Pre-MoCA, MoCA before moxibustion treatment.*

*^a^P-values were calculated with two-sample t-test.*

*^b^P-values were calculated with Chi-square test.*

*^c^P-values were calculated with Mann–Whitney U-test.*

*^d^Median (interquartile range).*

**TABLE 2:** Demographic and clinical conditions of two mild cognitive impairment groups.

Characteristic	TRUE (*n* = 30)	SHAM (*n* = 17)	*P*-value
Age (years)	64.3 ± 5.9	64.3 ± 7.3	0.998^a^
Gender (M/F)	10/20	4/13	0.480^b^
Education (years)	11 (11∼14)^d^	11 (8∼14)^d^	0.813^c^
Pre-MMSE	26 (25∼27)^d^	26 (25∼27)^d^	0.420^c^
Pre-MoCA	21.8 ± 2.6	21.9 ± 2.9	0.897^a^

*TRUE, true moxibustion group; SHAM, sham moxibustion group. Pre-MMSE, MMSE before moxibustion treatment; Pre-MoCA, MoCA before moxibustion treatment.*

*^a^P-values were calculated with two-sample t-test.*

*^b^P-values were calculated with Chi-square test.*

*^c^P-values were calculated with Mann–Whitney U-test.*

*^d^Median (interquartile range).*

**TABLE 3:** Mini-Mental State Examination and Montreal Cognitive Assessment scores before and after moxibustion treatment.

Characteristic	POST	PRE	*P*-value
**TRUE (*n* = 30)**			
MMSE	29.5 (28∼30)^a^	26 (25∼27)^a^	0.000^b^
MoCA	26.5 ± 2.6	21.8 ± 2.6	0.000^c^
**SHAM (*n* = 17)**			
MMSE	29 (28∼30)^a^	26 (26∼27)^a^	0.000^b^
MoCA	26.8 ± 2.1	21.9 ± 2.9	0.003^c^

*POST, after moxibustion treatment; PRE, before moxibustion treatment.*

*^a^Median (interquartile range).*

*^b^P-values were calculated with Wilcoxon signed-rank test.*

*^c^P-values were calculated with paired-sample t-test.*

### Resting-State Functional Magnetic Resonance Imaging Data Results

**FIGURE 2:**
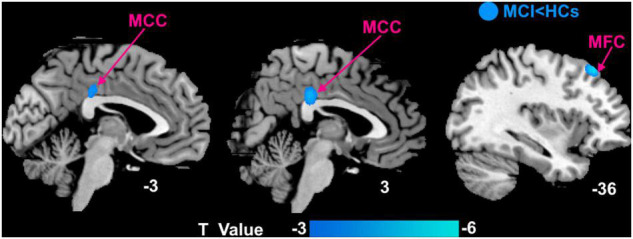
Voxel-wise comparison of DC between patients with MCI and HCs with the age, education, and gender as covariates. MCI, mild cognitive impairment; HCs, healthy controls; MFC, middle frontal cortex; MCC, middle cingulate cortex.

Compared with HCs, patients with MCI mainly had decreased DC in the left middle frontal cortex (MFC) and bilateral middle cingulate cortex (MCC) (Figure 2).

**FIGURE 3:**
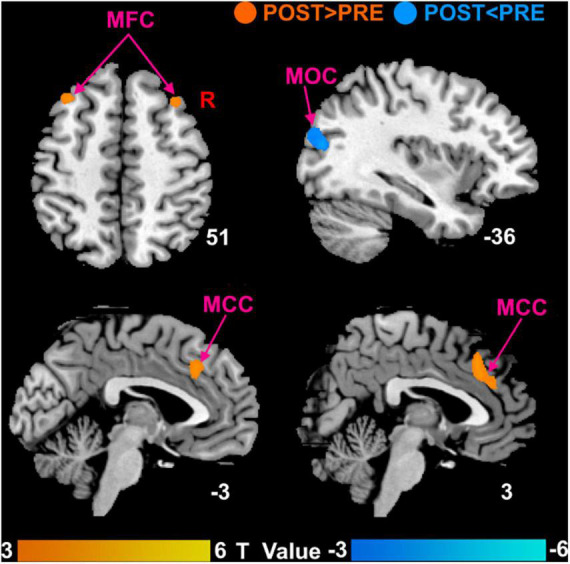
Brain responses to true moxibustion treatment. MFC, middle frontal cortex; MCC, middle cingulate cortex; MOC, middle occipital cortex; POST, patients with MCI after moxibustion treatment; PRE, patients with MCI before moxibustion treatment.

Compared with the baseline before treatment, patients with MCI of SHAM group after treatment showed no significant DC changes and patients with MCI of TRUE group after treatment mainly showed significantly increased DC in the bilateral MFC and bilateral MCC, as well as decreased DC in the left middle occipital cortex (MOC) (Figure 3). Unthreshold map showing voxels of any value is displayed in Supplementary Material.

**FIGURE 4:**
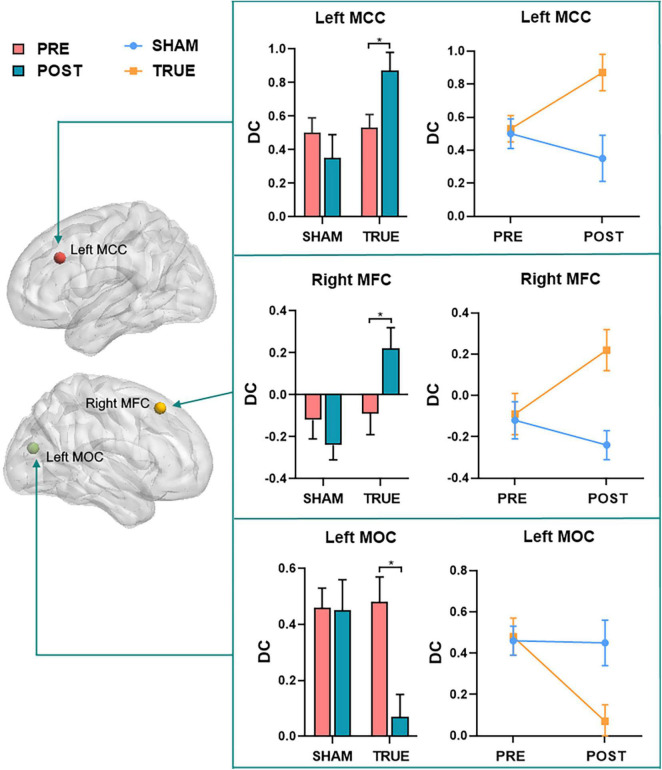
A *post hoc* analysis of regions (left MCC, right MFC, and left MOC) with significant interactions found by repeated measures ANOVA. MFC, middle frontal cortex; MCC, middle cingulate cortex; MOC, middle occipital cortex; POST, patients with MCI after moxibustion treatment; PRE, patients with MCI before moxibustion treatment; TRUE, true moxibustion group; SHAM, sham moxibustion group. The symbol * provided means that the difference is significant (*P* < 0.05).

Repeated-measures ANOVA showed that regions with significant interactions between the two groups of patients with MCI were left MCC (*F* = 4.684, *P* = 0.036), right MFC (*F* = 4.081, *P* = 0.049), and left MOC (*F* = 9.553, *P* = 0.003). A *post hoc* analysis was performed to find the average DC for each ROI, and resulting average DC for the two groups of data (SHAM and TRUE) is displayed in Figure 4.

**FIGURE 5:**
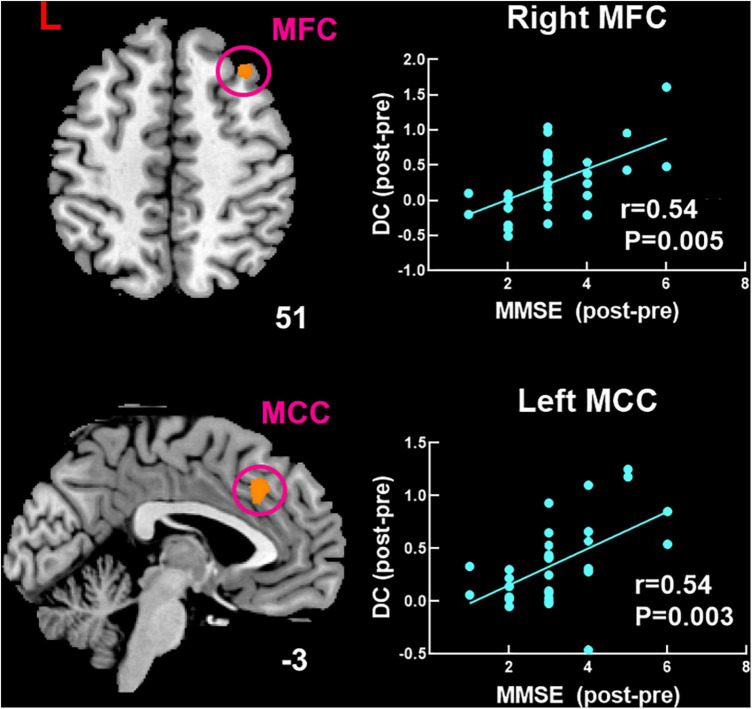
Significant correlation between changed DC and changed MMSE score after moxibustion treatment in patients with MCI of TRUE group. DC, degree centrality; MoCA, Montreal Cognitive Assessment; MMSE, Mini-Mental State Examination; MFC, middle frontal cortex; MCC, middle cingulate cortex.

The findings of correlation analysis are shown in Figure 5. Higher MMSE score was significantly positive correlated with increased DC in the right MFC (*r* = 0.54, *P* = 0.005) and left MCC (*r* = 0.54, *P* = 0.003) after moxibustion treatment.

## Discussion

In this study, we investigated potentially abnormal intrinsic connectivity of patients with MCI using the DC approach and assessed the effect of moxibustion treatment in patients with MCI. We found that (a) compared with HCs, patients with MCI mainly had decreased DC in the left MFC and bilateral MCC. (b) Both groups had significantly increased cognitive function as measured by MMSE and MoCA after moxibustion treatment. Patients with MCI of SHAM group showed no significant DC changes and patients with MCI of TRUE group were mainly found to have significantly increased DC in the bilateral MFC and bilateral MCC as well as decreased DC in the left MOC after moxibustion treatment. (c) Repeated-measures ANOVA showed that regions with significant interactions between the two groups of patients with MCI were left MCC, right MFC, and left MOC. In addition, higher MMSE score was significantly positively correlated with increased DC in the right MFC and left MCC after moxibustion treatment. In agreement with our hypothesis, the natural progression of the cognitive symptoms for patients with MCI was mitigated by moxibustion treatment.

### Alterations of Degree Centrality Patterns Between Patients With Mild Cognitive Impairment and Healthy Controls

In this study, we observed that patients with MCI had decreased DC in the left MFC and bilateral MCC. MFC is a critical target region in the progression of MCI and is involved in the executive function of the brain (Zheng et al., 2015), attention (Briggs et al., 2021), and episodic memory (Fujishima et al., 2014; Lau et al., 2016). The previous study has indicated that compared with HCs, patients with aMCI had decreased resting activity of left middle frontal gyrus (Li et al., 2015), which was related to changes of episodic memory. The deficit in episodic memory is one of the core cognitive impairments in MCI. MCC contained in the cingulate cortex is important region located in the medial cholinergic pathway, a major pathway in human cholinergic networks (Selden et al., 1998). In animal studies, the septocingulate pathway, in which cholinergic neurons of the basal forebrain innervate the cingulate cortex, is critically involved in the working/episodic memory of rats. A study has indicated that patients with MCI had decreased functional connectivity in the MCC (Qi et al., 2019), which related to memory capacity (Lin et al., 2017). These results are consistent with the one of our studies. In our study, the decreased DC in the MFC and MCC, thereby suggest that altered function of MFC and MCC is implicated in impairments in executive function and memory (especially delayed episodic memory), which is involved with being unable to recall previous things.

### Improvement of Patients With Mild Cognitive Impairment by Moxibustion Treatment

In this study, we found that moxibustion treatment improved MMSE and MoCA score in patients with MCI. MMSE contains five tests: orientation, memory, calculation and attention, recall, and language. MoCA includes visual space, executive function (clock drawing), naming, attention, language, abstract ability, memory, and orientation. After moxibustion treatment, the memory, executive ability and attention were significantly improved in patients with MCI of TRUE group, and the most obvious was the improvement of memory in this study. Although the total MMSE and MoCA score in patients with MCI of SHAM group also improved significantly, the improvement in the above three sub-items was not as good as that in patients with MCI of TRUE group. It has been reported that moxibustion treatment is one of the reliable clinical treatments and improves the level of cognitive function in patients with MCI [refer to the reviews from Wang et al. (2020) and Zhang et al. (2020)], which was consistent with our findings. Hence, our study provides new evidence to support that moxibustion treatment may indeed be particularly effective in improving cognitive performance in patients with MCI.

In this study, we found increased DC in the bilateral MFC in patients with MCI of TRUE group after moxibustion treatment and a significantly positive correlation between increased DC in the right MFC and higher MMSE score. MFC, a significant cortical region, is an important component of the dorsolateral prefrontal cortex and is contained in the executive control network (ECN) that is related to executive function, attention, and working memory (Zheng et al., 2015; Briggs et al., 2021). Executive function is an advanced form of cognitive function, which controls and regulates other cognitive processes when completing complex cognitive tasks. Executive function plays a crucial role in cognitive field. A body of study has indicated that functional brain activity within portions of the ECN is abnormal with patients with MCI (Firbank et al., 2016). It has been demonstrated that for patients with MCI, the decreased frontoparietal network is mainly due to the memory-retrieval tasks (Stretton and Thompson, 2012), perhaps reflecting worse self-monitoring in MCI individuals (Li et al., 2015). An interesting case study has showed that the right MFC may play a role in the shifting of attention from exogenous to endogenous control (Lin et al., 2017). In our study, we found that patients with MCI had decreased DC compared with HCs and increased DC after moxibustion treatment in the MFC. Therefore, moxibustion treatment is likely to improve execution, memory and attention by regulating the internal connection of MFC.

We also found that increased DC in the bilateral MCC in patients with MCI of TRUE group after moxibustion treatment and a significantly positive correlation between increased DC in the left MCC and higher MMSE score. MCC is the neural substrates of the medial cholinergic pathway, and the cholinergic pathway is in a position to promote memorability of novel and motivationally relevant events (Selden et al., 1998). It has been indicated that networks disruption of MCC is associated with memory deficits and cognitive decline in dementia (Seeley et al., 2009; Li et al., 2012; Xie et al., 2012). Moreover, Li et al. (2012) has found that the regional cerebral blood flow in subjects with mild AD after donepezil treatment is significantly increased in the MCC. In our study, we found that patients with MCI had decreased DC compared with HCs and increased DC after moxibustion treatment in the MCC. Therefore, we speculate that the increased connectivity after moxibustion treatment may be interpreted as the improvement toward normalcy. Moreover, during our moxibustion treatment, the warm stimulation makes patients with MCI feel warm, satisfied, and active, which may be improvement of the patient’s attention and may mediate moxibustion-induced influences on associated brain regions, such as MCC sensitive to pain caused by thermal stimulation (Vogt, 2005).

In addition, we found increased DC in the left MOC in patients with MCI of TRUE group after moxibustion treatment. MOC is located in the primary visual cortex and is involved in processing visual recognition. Studies have demonstrated that functional connectivity in the middle occipital gyrus is altered in patients with aMCI. For instance, Cai et al. (2017) have reported increased connectivity between right lingual gyrus and left middle occipital gyrus in the visual network. In another study, left middle occipital gyrus also have increased functional connections with fusiform gyrus in patients with aMCI, that is not a coincidence, and may imply that the middle occipital gyrus plays a critical role in visual cognition, especially face recognition. Golby et al. (2005) have found that patients with AD also have a spared activation for novel relative to familiar scenes in the left middle occipital gyrus. Thereby, our findings suggest that moxibustion treatment may improve cognitive function by enhancing the intrinsic function of the MOC and further normalizing abnormal hyperactivity of the MOC, as indicated by decreased DC.

Interestingly, in a systematic review of animal studies by Choe et al. (2018), the efficacy of moxibustion treatment in preventing cognitive impairment has been validated and its underlying mechanism has been clarified to a certain extent. For instance, moxibustion treatment may prevent cognitive impairment by inhibiting neuronal apoptosis to prevent neuronal loss. Moxibustion treatment can also reduce the risk of dementia by controlling inflammation. But whether altered DC after moxibustion treatment in patients with MCI is related to these mechanisms needs further to research.

There were several limitations that should be noted. First, our findings might be limited by the relatively small sample size, and future studies with larger sample sizes are needed to validate our results. Second, we did not have an index to access and quantify patients’ expectations during moxibustion treatment sessions. We cannot rule out the non-specific effect of moxibustion treatment; therefore, further studies need to quantify patients’ expectations and explore the effect of moxibustion on the clinical efficacy and physiological mechanism of some non-specific factors during long-term moxibustion treatment.

## Conclusion

We found that compared with HCs, patients with MCI had decreased DC in the left MFC and bilateral MCC, which were implicated in impairments in executive function and memory. More importantly, there was difference between SHAM and TRUE groups in the changes of DC findings after moxibustion treatment. Patients with MCI of SHAM group had no significant DC findings after 2-month treatment. Patients with MCI of TRUE group were mainly found to have significantly increased DC in the bilateral MFC and MCC, as well as decreased DC in the left MOC after 2-month treatment. It was found that the improvement level of cognitive function as measured by MMSE and MoCA was significantly related to changed DC in patients with MCI of TRUE group after moxibustion treatment. Moxibustion treatment may improve cognitive function by enhancing the intrinsic function of the MFC, MCC, and MOC. These findings demonstrate the potential value of moxibustion treatment in preventing the progression of MCI.

## Data Availability Statement

The original contributions presented in this study are included in the article/Supplementary Material, further inquiries can be directed to the corresponding authors.

## Ethics Statement

The studies involving human participants were reviewed and approved by the Ethics Committee, First Affiliated Hospital, Guangxi University of Chinese Medicine. The patients/participants provided their written informed consent to participate in this study.

## Author Contributions

PL and DD were responsible for the study concept and design. YW, LZ, WM, and LL contributed to acquisition of MRI data. KX, CL, BG, SZ, and XZ assisted with data analysis and interpretation of findings. PL and KX drafted the manuscript. All authors critically reviewed the content and approved the final version for publication.

## Conflict of Interest

The authors declare that the research was conducted in the absence of any commercial or financial relationships that could be construed as a potential conflict of interest.

## Publisher’s Note

All claims expressed in this article are solely those of the authors and do not necessarily represent those of their affiliated organizations, or those of the publisher, the editors and the reviewers. Any product that may be evaluated in this article, or claim that may be made by its manufacturer, is not guaranteed or endorsed by the publisher.

## Footnotes

1
http://www.fil.ion.ucl.ac.uk/spm

2
http://rfmri.org/dpabi
