# Supplementary figures and images for: Effect of Moxibustion Treatment on Degree Centrality in Patients With Mild Cognitive Impairment: A Resting-State Functional Magnetic Resonance Imaging Study
Source: Front Hum Neurosci. 2022 Aug 2;16:889426. doi: 10.3389/fnhum.2022.889426 (PMC9378775; doi:10.3389/fnhum.2022.889426)

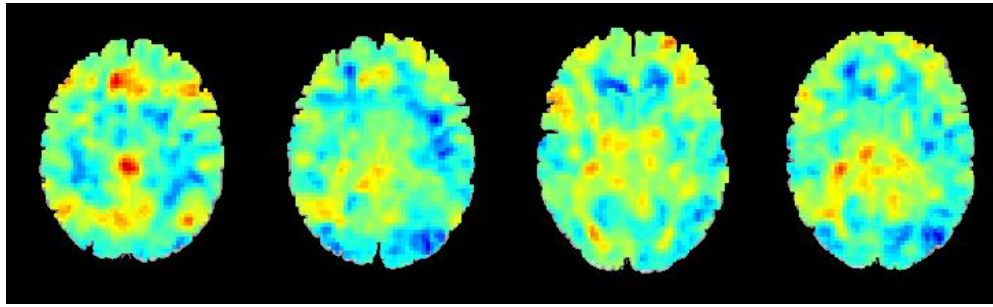

Unthreshold map showing voxels of any value

Supplement: Supplementary file 1 [file Data_Sheet_1.pdf]
